# Arabic Sentiment Classification Using Convolutional Neural Network and Differential Evolution Algorithm

**DOI:** 10.1155/2019/2537689

**Published:** 2019-02-26

**Authors:** Abdelghani Dahou, Mohamed Abd Elaziz, Junwei Zhou, Shengwu Xiong

**Affiliations:** ^1^School of Computer Science and Technology, Wuhan University of Technology, 122 Luoshi Road, Wuhan, Hubei 430070, China; ^2^Department of Mathematics, Faculty of Science, Zagazig University, Zagazig, Egypt

## Abstract

In recent years, convolutional neural network (CNN) has attracted considerable attention since its impressive performance in various applications, such as Arabic sentence classification. However, building a powerful CNN for Arabic sentiment classification can be highly complicated and time consuming. In this paper, we address this problem by combining differential evolution (DE) algorithm and CNN, where DE algorithm is used to automatically search the optimal configuration including CNN architecture and network parameters. In order to achieve the goal, five CNN parameters are searched by the DE algorithm which include convolution filter sizes that control the CNN architecture, number of filters per convolution filter size (NFCS), number of neurons in fully connected (FC) layer, initialization mode, and dropout rate. In addition, the effect of the mutation and crossover operators in DE algorithm were investigated. The performance of the proposed framework DE-CNN is evaluated on five Arabic sentiment datasets. Experiments' results show that DE-CNN has higher accuracy and is less time consuming than the state-of-the-art algorithms.

## 1. Introduction

People and organizations are posting their information and opinions on various social media platforms such as Twitter and Facebook. Understanding public sentiments, emotions, and concerns expressed on these platforms is a crucial issue, which is the interest of sentiment analysis (SA). SA is a natural language processing (NLP) application that focuses on automatically determining and classifying the sentiment of large amounts of text or speech [1, 2]. Arabic is a Semitic language known by its morphology richness and different written and spoken forms such as modern standard Arabic (MSA) and its various dialects. Arabic morphology and structure complexity create many challenges such as the shortage of large datasets and limited tools to perform sentiment analysis [3, 4]. Even deep neural networks (DNNs) [5] and convolutional neural networks (CNNs) [6] have shown promising and encouraging performance, little research on sentiment analysis using deep learning (DL) techniques has been done for Arabic language [7–9] while many researches have been done on other languages [10–12]. Research on Arabic language using deep-learning techniques is still relatively scarce which is worth to be investigated.

To choose the best architecture and hyperparameters for a DL model and apply it to Arabic sentiment classification, the model is usually evaluated on different architectures and hyperparameter combinations manually or using previous successful models directly [13]. Moreover, the building task of a DL model for SA remains a very crucial process that requires the involvement of specialists in the domain and language or the integration of feature engineering techniques. In addition, designing a DL model is still a complex and time-consuming task. The assessment of DL models parameters requires a fitting and evaluation process on the test data, which can be very expensive and infeasible on small computing units. Therefore, an effective methodology to select the best architecture for a DL model with optimal hyperparameters is needed to build a successful Arabic sentiment classification system. A lot of work has been done in order to evolve DL models using NeuroEvolution (NE) methods [14] on different tasks such as image classification [15] using particle swarm optimization (PSO), and handwritten digit recognition based on genetic algorithms and grammatical evolution [16]. In the same context, this paper presents an alternative NE approach for Arabic sentiment classification using the differential evolution (DE) [17]. The DE algorithm is adopted since it is known by its remarkable performance using different mutation strategies in several literatures, as well as, it has less parameters to fine-tune. To the best of our knowledge, this is the first work that attempted to address the problem of automatically building a deep neural network model for Arabic sentiment classification using DE algorithm. The proposed DE-CNN model focuses on utilizing DE algorithm to automatically find and tune appropriate parameters to build the optimal CNN model. Since CNN have been applied extensively to sentiment classification on other languages, Arabic sentiment classification is chosen as a well-known and widely used task, which constitutes a good environment to validate and evaluate the performance of DE-CNN.

DE-CNN starts by generating a population, where each individual represents a configuration selected randomly from each parameter possible values. Then, DE-CNN evaluates each individual through computing fitness function value using the current configuration. After that, all individuals in the population are updated using DE algorithm operators. These steps are repeated until the terminal criteria are satisfied. To evaluate the performance of the proposed framework, various Arabic sentiment classification datasets covering Twitter data are used. The evaluations on these datasets show that the proposed framework outperformed existing methods.

The main contributions of this paper can be summarized as follows:

- Modeling the problem of evolving CNNs as a metaheuristic optimization task to build an Arabic sentiment classification system
- Using two different fitness evaluation techniques to assess the generalization of the CNN
- Integrating two different mutation strategies to improve the exploration and exploitation ability of DE algorithm
- Building and training different CNN architectures with variable number of parallel convolution layers

The rest of this paper is organized as follows: Section 2 provides related works with respect to Arabic sentiment classification and NE.  Section 3 describes the fundamentals of DE algorithm and CNN. The proposed framework is introduced in Section 4.  Section 5 presents the evaluation of the proposed framework, while Section 6 gives the conclusion and presents our future work.

## 2. Related Work

In this section, we will review the most recent works related to Arabic sentiment classification and NE. Recently, many works have been conducted on SA targeting English, and other European languages. However, a small number of researches focus on the Arabic language [18, 19] using DL models. Sallab et al. [20] trained several DL models as described in their original papers for Arabic sentiment classification including deep neural networks (DNNs), deep belief networks (DBNs), deep autoencoder (DAE), and recursive autoencoder (RAE). Al-Azani et al. [21] investigated the problem of imbalanced datasets for Arabic sentiment polarity determination. They conducted a comparison between different traditional machine learning classifiers and ensembles such as k-nearest neighbor (k-NN), support vector machines (SVMs), voting, bagging, boosting, stacking, and random forests. Moreover, Al-Azani et al. [22] conducted an empirical evaluation of two state-of-the-art DL models, which are unidirectional and bidirectional Long Short-Term Memory (LSTM) and its simplified variant Gated Recurrent Unit (GRU), to detect sentiment polarity of Arabic microblogs. Alayba et al. [23] used DNN and CNN alongside several Machine Learning algorithms to perform Arabic SA on health services. In their experiments, they reported that the best classifiers were SVM and stochastic gradient descent (SGD) in which they did not investigate the effect of DL models architecture and parameters.

NE is considered as a subfield within artificial intelligence (AI). It aims to automatically evolve neural networks architectures and hyperparameters based on the use of evolutionary algorithms. For example, Young et al. [24] presented a framework named multinode evolutionary neural networks for deep learning (MENNDL) to learn optimized CNN hyperparameters via a genetic algorithm (GA). Restricting convolutional layer to three layers, hyperparameters such as filter size and the number of filters for each convolutional layer were optimized. Verbancsics and Harguess [25] proposed a modification of hypercube-based NeuroEvolution of augmenting topologies (HyperNEAT) [26] to evolve a CNN for image classification task. The methodologies were evaluated on MNIST dataset. Tirumala et al. [27] studied the feasibility of using evolutionary approaches to propose the prospects of evolving deep architectures with the aim of reducing training time of DNNs. By evaluating their approach on MNIST dataset, the training time of DNN was accelerated over the regular approach with a time difference of over 6 hours. Based on reinforcement learning, Baker et al. [28] proposed a metamodeling algorithm named MetaQNN. For a learning task such as image classification, MetaQNN is used to automate the generation of CNN architectures and tested over MNIST dataset. Loshchilov and Hutter [29] proposed an alternative deep neural network hyperparameters optimization instead of the grid search, random search, or Bayesian optimization. Covariance Matrix Adaptation Evolution Strategy (CMA-ES) was used to evolve several hyperparameters in the optimizer, convolution, and fully connected layers. Based on Cartesian genetic programming, Suganuma et al. [30] presented a work to perform image classification on the CIFAR-10 dataset by automatically building and optimizing CNN architectures. Their core of research was focusing on convolution blocks and tensor concatenation, and they do not consider dense layers or hyperparameters optimization. They automatically generated competitive CNN architectures that can compete with the state-of-the-art networks. Xie and Yuille [31] have adopted genetic algorithms (GAs) to evolve CNN architectures by proposing a binary encoding method to represent GA individuals in a fixed-length string. They used two common datasets such as MNIST and CIFAR-10 to perform visual recognition and evolve the CNN architectures based on recognition accuracy. Following the same working principles as NEAT [32], Miikkulainen et al. [33] introduced an automated approach for evolving deep neural networks named Cooperative DeepNEAT (CoDeepNEAT), which learn complex convolutional, feedforward, and recurrent layers to evolve the network architecture. Real et al. [34] introduced a technique based on GAs to generate a fully trained neural network that does not require any postprocessing.

## 3. Preliminaries

### 3.1. Differential Evolution

The differential evolution (DE) is one of the most popular evolutionary algorithms introduced by Storn and Price in [17, 35]. DE has been used in different optimization tasks such as computer vision [36, 37] and text classification [38]. The DE starts by initializing training parameters such as population size *N*, individual dimension *N*_par_, mutation scaling parameter *F*, and crossover probability *CR*. At the beginning, a population *X* of size *N* and dimension *N*_par_ is generated using

(1) $$\begin{array}{c} x_{i}=L_{i}+\text{rand}\left(N,N_{\text{par}}\right)*\left(U_{i}-L_{i}\right),\;x_{i}\in X,\;\;i=1,2,\ldots ,N, \end{array}$$

where *L* and *U* represent lower and upper boundaries of the search space, respectively. rand(.,.) is the function used to generate a random matrix in the interval [0,1].

*Mutation operator* is used to create a new individual *v*_*i*_ from the current parent individual *x*_*i*_. DE scheme (or DE/rand/bin) defined in Equation (2) performs the mutation operation.

(2) $$\begin{array}{c} v_{i}^{t}=x_{r1}^{t}+F*\left(x_{r2}^{t}-x_{r3}^{t}\right), \end{array}$$

where *x*_*r*1_, *x*_*r*2_, and *x*_*r*3_ are different individuals randomly chosen from the population at iteration *t*.

*Crossover operator* is used to generate an offspring individual from *v*_*i*_ and *x*_*i*_ as the following:

(3) $$\begin{array}{c} z_{ij}^{t}=\left\{\begin{array}{cc} v_{ij}^{t}, & \text{if}\;\;\gamma_{j}\leq CR\text{ or }\delta_{i},\\ x_{ij}^{t}, & \text{otherwise,} \end{array}\right. \end{array}$$

where *γ*_*j*_ is a random value chosen for the *j*th decision variable and *δ*_*i*_ represents a random decision variable index taken from [1, *N*_par_].

Then, the fitness function fit_*x*_*i*__ of the parent individual *x*_*i*_ and the fitness function fit_*z*_*i*__ of the offspring *z*_*i*_ are computed.

*Selection operator* is used to select the best individual from the parent individual *x*_*i*_ and the offspring *z*_*i*_ based on the calculated fitness function values as defined in the following equation:

(4) $$\begin{array}{c} x_{i}^{t+1}=\left\{\begin{array}{cc} z_{i}^{t}, & \text{if fi}{\text{t}}_{z_{i}}\leq \text{fi}{\text{t}}_{x_{i}},\\ x_{i}^{t}, & \text{otherwise}. \end{array}\right. \end{array}$$

The previous steps are repeated until the stop condition is met. If it is satisfied, the DE stops and returns the best individual. Otherwise, it will continue by starting again from mutation phase. DE algorithm can use different strategies to perform mutation, where some of them are used to improve the exploration and exploitation ability of the search space [39, 40]. These strategies can be distinguished through using the representation “DE/a/b” where “DE” refers to the differential evolution, “a” indicates the solution to be mutated, and “b” represents the number of different solutions used. In this paper, only two strategies are used where the first one is the “DE/best/1” given as

(5) $$\begin{array}{c} v_{i}^{t}=x_{b}^{t}+F*\left(x_{r2}^{t}-x_{r3}^{t}\right), \end{array}$$

whereas the second one is “DE/best/2” given as

(6) $$\begin{array}{c} v_{i}^{t}=x_{b}^{t}+F*\left(x_{r2}^{t}-x_{r3}^{t}\right)+F*\left(x_{r3}^{t}-x_{r4}^{t}\right), \end{array}$$

where *x*_*b*_^*t*^ represents the best solution at the iteration *t*.

### 3.2. Convolutional Neural Network

**Figure 1:**
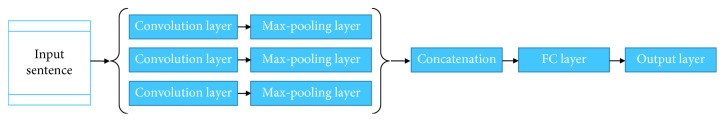
The parallel CNN architecture.

Deep learning approaches known by their ability to automatically learn features have shown remarkable performance in various fields. For example, computer vision (CV) [41], speech recognition [42, 43], NLP [44, 45], and a large variety of applications [46]. In this section, a common deep learning model named parallel convolutional neural network (CNN) for sentence classification is described.  Figure 1 shows the parallel CNN architecture where the CNN model consisting of one-dimension parallel convolution layers (1D-CNN) is used to capture local semantic features by using a unique filter size at each parallel convolutional layer [44]. To select global semantic features, a one-dimension pooling layer is implemented at the end of each convolution layer. The outputs from pooling layer are concatenated and fed to a fully connected (FC) layer. Finally, an FC layer with sigmoid or Softmax acts as an output layer, which is used to produce the classification results based on the inputted features from previous layers. CNN is known by its convolution operation that uses filters, where each filter can learn to produce a feature map. At the same layer in CNN, same filter weights are shared. CNN takes input as a matrix that represents a sentence, where each row is *S*_*d*_ dimensional vector assigned to a specific word from the sentence. These word vectors are build using a neural language model (NLM) such as word2vec [47, 48] which represents the semantic relations between words as vectors. As an example, if we assume that the input sentence has 20 words and each word is represented as a *S*_*d*_=100 dimensional vector, then the size of the input layer of the CNN will be 1 × 20 × 100. To address the problem of overfitting, layers such as pooling and dropout are commonly used. For the convolution and fully connected layers, Sigmoid, Hyperbolic Tangent (tanh), and Rectifier (ReLU) [49] are activation functions which can be applied in neural networks.

## 4. Proposed Framework

In this section, the proposed DE-CNN framework based on DE algorithm for evolving CNN will be presented in detail. The aim of the proposed DE-CNN is to determine the optimal architecture and parameters for a CNN, and enhance the performance of Arabic sentiment classification. To achieve this goal, the DE algorithm is used to search for the best configuration from a set of parameters used to build and train a CNN. Unlike the most existing CNN architectures for text classification that employ one dimension (1D) convolution and pooling operations, we apply 2D convolution operations in DE-CNN. To gain better performance, words from the dataset are inputted to the CNN as a word-embedding matrix with two dimensions, where each word is represented by a vector extracted from a pretrained word embedding. Therefore, the 2D convolution operations may help to extract more meaningful sentiment features and prevent destroying the structure of the word embeddings [50].

The proposed DE-CNN framework consists of three stages: initialization, evaluation, and update. Firstly, the initialization stage, DE-CNN starts by generating a random population *X* with size *N* and dimension *N*_par_. Where *N*_par_ represents the number of hyperparameters used to control the configuration of CNN such as the number of convolution filters, convolution filter size, number of filters per convolution filter size (NFCS), number of neurons in fully connected (FC) layer, and dropout rate. Each parameter contains a list of different values, where a random selected value is used to initialize each solution *x*_*i*_, (*i*=1,2,…, *N*) in *X*. Moreover, two fitness evaluation techniques were adopted to divide the dataset into training and testing such as random train/test split with 80% for training and 20% for testing (80/20) or k-fold cross validation. Secondly, the evaluation stage starts by building the CNN based on the current *x*_*i*_, where the number of convolution filters determines the number of parallel convolution layers. Each convolution filter size will be assigned to a parallel convolution layer followed by a max-over time pooling layer, which is used to reduce the dimensionality and computation cost. Pooling size is represented as (max(*S*) − *fz*+1,1), where *S* is the sequence length and *fz* is the filter size assigned to the previous convolution layer. A concatenation operation is performed to merge the outputs from each pooling layer and fed to the FC layer. Moreover, DE-CNN builds a hidden layer followed by a dropout operation based on the corresponding values from *x*_*i*_. After building the CNN using *x*_*i*_, the testing set is used to evaluate the performance of the CNN model through the fitness function value fit_*i*_ of the current solution *x*_*i*_. After that, DE algorithm selects the best solution *x*_*b*_ having the highest fitness value fit_*b*_. Finally, in the updating stage, the solutions of the population *X* are updated using the operators of DE algorithm crossover, mutation, and selection. Evaluation and update stages are repeated until the stop condition is met. The three stages of the proposed DE-CNN framework are described with more details in the following sections.

### 4.1. Initialization Stage

In this stage, the list of values corresponding to each parameter is generated and DE algorithm parameters such as crossover and mutation are set. Moreover, the size of solutions *N* and the maximum number of iterations *t*_max_ are chosen. Then a random integer population *X* with size *N* and dimension *N*_par_ is generated using the following equation:

(7) $$\begin{array}{c} x_{ij}=l_{j}+\text{rand}*\left(u_{j}-l_{j}\right),\;j=1,2,\ldots ,N_{\text{par}},\;\;i=1,2,\ldots ,N, \end{array}$$

where *L*_*j*_ and *U*_*j*_ represents the lower and upper boundaries of the *j*th parameter of *x*_*i*_ ∈ *X*, receptively.  Table 1 lists an example solution *x*_*i*_=[2, 4, 3, 1, 5] that represents a configuration to build a random CNN model. Each index value in *x*_*i*_ vary in a range of minimum and maximum values as described in Table 1 where each index corresponds to a random parameter value *x*_1*j*_.

**Figure 2:**
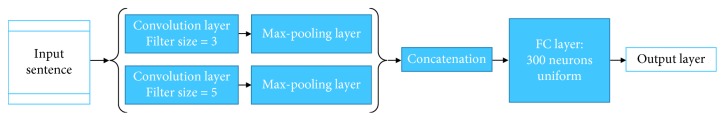
CNN architecture and parameters for the example listed in Table 1.

As shown in Table 1, filter sizes list defines the parallel architecture of the CNN model. The number of values in the list will define the number of parallel convolution layers, where each value refers to the convolution filter size applied when performing convolution operation. Each convolution filter size will have 150 distinct filters with the same size and different initialization values. Pooling layer with the max-pooling operation is placed after each convolution layer which will take the same filter size value to calculate the pooling size. A concatenation operation is applied to merge the output feature vectors from each pooling layer to be fed into the FC layer. FC layer consists of 300 neurons initialized using the uniform mode as shown in Figure 2. An output layer used to produce the classification accuracy is implemented at the end of the generated CNN model.

### 4.2. Evaluation Stage

This stage starts by constructing the CNN model based on the parameters of the current solution *x*_*i*_. The 80/20 split method or 5-fold CV method are used to evaluate the fitness function fit_*i*_ for each *x*_*i*_. Where 80/20 split method randomly selects 80% of the data as training set and 20% as testing set with once evaluation.

Meanwhile, in 5-fold CV, the dataset is divided into different five groups, where four of them are used to represent the training sets and one of them represents the testing set. The evaluation is repeated five times, and the average of the classification accuracy over the five runs is used as the fitness function value fit_*i*_:

(8) $$\begin{array}{c} {\text{fit}}_{i}=\frac{\sum_{k=1}^{5}{\text{acc}}_{k}}{5}, \end{array}$$

where acc_*k*_ represents the accuracy of classification of the *k*th run.

### 4.3. Update Stage

In this stage, the best solution *x*_*b*_ with the highest fitness function value fit_*b*_ is determined. Then each solution *x*_*i*_ in the current population *X* is updated using the three operators of the DE algorithm as mentioned in Section 3.1. The evaluation and update stages are repeated until the stop condition is met. Here, the maximum number of iterations (*t*_max_) is used as the stop condition.

**Figure 3:**
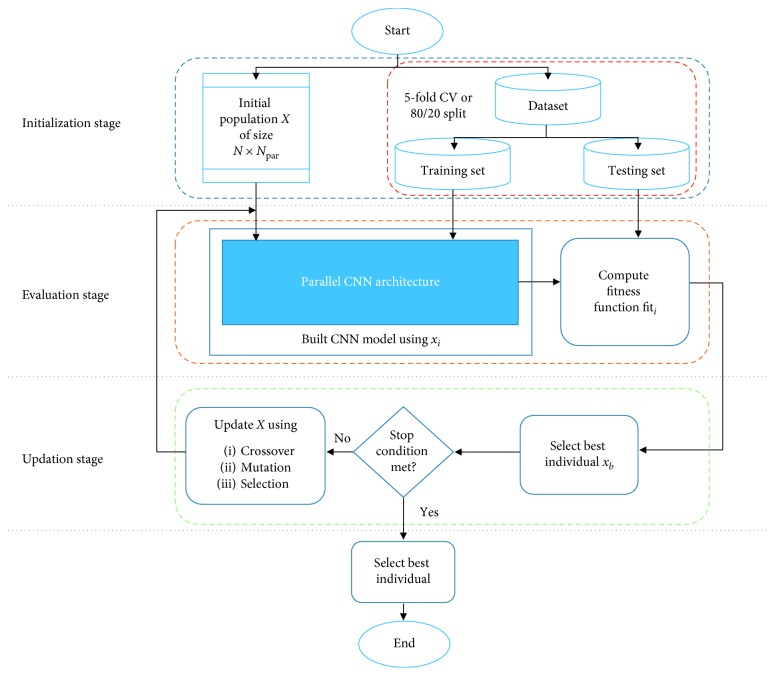
The proposed DE-CNN framework.

The general framework of the proposed model DE-CNN is shown in Figure 3.

## 5. Experimental Results

Several experiments are conducted using different datasets for Arabic sentiment classification with their balanced and imbalanced shapes, where each dataset is described in Section 5.1. CNN parameter settings are described in Section 5.2. Performance measures are discussed in Section 5.3, while, Sections 5.4to 5.9 present various experimental series to evaluate the performance of the proposed framework.

### 5.1. Sentiment Classification Datasets

In this section, various Arabic sentiment datasets used to evaluate the proposed framework are introduced. Nabil et al. [18] presented the labeled Arabic sentiment tweets dataset (ASTD) for subjectivity and sentiment polarity classification. ASTD contains over than 10,000 Arabic tweets classified as objective, subjective positive, subjective negative, and subjective mixed. In our experiments, we used the balanced shape of the dataset (ASTD-B) where the maximum number of training tweets is equal to the number of tweets in the minority class. Mohammad et al. [51] proposed the Syrian tweets Arabic sentiment analysis (STD) dataset consisting of 2000 tweets from Syria. Alomari et al. [52] collected the Arabic Jordanian general tweets (AJGT) corpus written in Jordanian dialect and modern standard Arabic in May 2016. AJGT consists of manually annotated 900 positive tweets and 900 negative tweets. Abdulla et al. [53] collected the Twitter dataset for Arabic sentiment analysis (ArTwitter), where the annotation of the dataset is performed by two human experts. We used same ArTwitter dataset evaluated in [7] consisting of almost 2000 labeled tweets. Aziz et al. [7] used multiple Twitter datasets, namely, ASTD, ArTwitter, and QRCI, to build a balanced dataset and evaluate their system for sentiment classification. We will refer to this dataset that consists of more than 4000 tweets as AAQ in our study. All used datasets in our experiments are in a balanced shape except STD dataset, since STD is a highly imbalanced dataset.

Each dataset in our experiments has been preprocessed after applying several actions to clean and normalize the Arabic text. Stopwrods has been removed after mapping each word from the dataset vocabulary to a stopwords list that contains 750 most frequent Arabic words (https://github.com/mohataher/arabic-stop-words). Punctuations, numbers, symbols, and non-Arabic words have been replaced by keywords such as PUNK, NUM, and UNK. We conduct our experiments on nondiacritized Arabic text, and for that all diacritics were deleted. Arabic characters such as Alef, Teh Marbuta, and ALEF Maksura are normalized.

### 5.2. CNN Architecture and Parameters

In this section, we will learn the proper CNN architecture and parameters automatically using differential evolution (DE) algorithm. Parameterizing the CNN model using DE requires an individual structure (configuration). In our experiments, the individual structure consists of parameters from two layers which are the convolution layer and FC layer. In addition, only three convolution layers are set to be trained in parallel at the same time at max. In total, five different parameters are coded into each individual. We fix the optimizer and merge operation for each individual, and we change the parameters values to maximize the accuracy of the classified sentences over a test set. Moreover, CBOW58 Arabic word embeddings from [54] used to transform datasets into vector representations as an input to the CNN.  Table 2 lists the possible values for each parameter.

The number of filters used to perform convolution operation varies from 50 to 500 filters per filter size. The number of convolution layers trained in parallel is related to the dimension of filter sizes list. A random function is implemented to generated random filter sizes list, where each filter size can have a value that ranges from 2 to 9. The maximum number of generated filter sizes in a list is limited to three filter sizes. For example, if the generated filter sizes list is [2, 5, 7], that means three convolution layers are running in parallel, where each one of the layers uses a single filter size from the generated list. The number of neurons used to construct the fully connected layer is 50, 100, 200, 300, 350, 400, or 500. ReLU function is used as an activation function for both convolution and FC layer. Different initialization modes for the FC layer were investigated such as uniform, LeCun uniform, normal, and He uniform. To prevent the overfitting of the CNN, a regularization technique named Dropout is adopted with different rates that range from 0.2 to 0.9 and used in three positions: after embedding layer, pooling layer, and FC layer. Adam is used as an optimizer to train the CNN. Moreover, to handle sentences with variable lengths, all sentences are padded (zero-padding is adopted) so they all become of a length equal to the maximum sentence length in each dataset.

### 5.3. Performance Measures

A set of measures such as precision, recall, accuracy, and F-score were used to evaluate the performance. These measures are defined as follows:

(9) $$\begin{array}{c} \text{precision}=\frac{\text{TP}}{\text{TP}+\text{FP}}\times 100,\\ \text{recall}=\frac{\text{TP}}{\text{TP}+\text{FN}}\times 100,\\ \text{accuracy}=\frac{\text{TP}+\text{TN}}{\text{TP}+\text{TN}+\text{FP}+\text{FN}}\times 100,\\ \text{F}‐\text{score}=2\times \frac{\text{precision}\times \text{recall}}{\text{precision}+\text{recall}}, \end{array}$$

where TP, TN, FP, and FN denote true positive, true negative, false positive, and false negative, respectively. Experiments have been carried out on a machine with GeForce GTX 1080 Ti graphic card and 126 Gb of RAM.

It is worth mentioning that 5-fold CV and 80/20 dataset split techniques were used only in the DE algorithm evaluation stage to select the optimal configuration for each dataset. If 80/20 dataset split is used, we evaluate the best configuration again using 5-fold CV and we report the 5-fold CV classification accuracy in our tables to assess the generalization of the selected configuration, whereas 10-fold CV was used to calculate the final classification result for each optimal configuration and compare it with state-of-the-art methods, where most of them use 10-fold CV for evaluation. However, to avoid the bias in the validity of the results when using different data splitting strategies (i.e., 5-CV, 10-CV or 80/20 split), a new model is created with new weights initialization for each fold or evaluation stage. This means that the CNN will be trained from scratch for each selected configuration and dataset split. Besides, during the evaluation stage in the DE algorithm, if 80/20 split is chosen to calculate the fitness function, each time a new model is created with new weights where 80% samples from the dataset are used for training and 20% samples for testing. To evaluate the optimal configuration obtained from searching using 80/20 split using 5-CV or 10-CV, a new CNN is created and trained from scratch for each evaluation iteration with the same parameters and new weights as mentioned earlier. For that, each trained CNN is fitted and tested on unseen samples.

### 5.4. Experimental Series 1: Influence of DE Algorithm Parameters

In this experimental series, we analyze the influence of different parameters of DE algorithm, which include population size, mutation parameter *F*, and DE strategy (i.e., DE/best/1, DE/best/2) defined using Equations (5) and (6).  Table 3 lists the results of the proposed DE-CNN with the following DE parameters: population of sizes 5 or 10, DE/best/1 as DE strategy, and *F* is set to 0.3 or 0.7. From this table, it can be observed that the accuracy with population size 10 is better than the accuracy with population size 5. On the contrast, ArTwitter dataset gets less accuracy when population size changes from 5 to 10. However, the proposed DE-CNN takes more time if population size is equal to 10.

After that, the value of *F* is changed to 0.7 with the same strategy and population size. In this case, it can be seen that the classification accuracy at a population size 5 is better than accuracy when using a population of size 10 with *F*=0.3, but with less computational time. Moreover, the average classification accuracy of the proposed model at population of size 10 and *F*=0.7 is the best as shown in Table 3.

The effect of DE strategy is tested through using DE/best/2 as given in Table 4, where *F* is set to 0.3 or 0.7 and the population size is set to 5 or 10. The reported time in all tables is in seconds. As shown in Table 4, the classification accuracy at population size equal to 5 and *F*=0.3 is better than all the results of the previously used DE strategy (i.e., DE/best/1). However, the computation time is shorter. With DE/best/2, population size 10, and *F*=0.3, the best average accuracy across all datasets and the shortest time can be obtained. In this experimental series, the reported time has been calculated during the process of selecting the optimal configuration using 5-fold CV as the fitness evaluation technique for each individual.

From all results listed in Tables 3 and 4, we can select the optimal configuration for the proposed model that balances the accuracy and time. The best DE parameters are *F*=0.3, *CR*=0.2, strategy DE/best/2, and population size equal to 10. The proposed DE-CNN model based on these DE algorithm parameters can be named as DE-CNN-5CV model.  Table 5 shows the optimal configuration for each dataset produced using DE-CNN-5CV model.

### 5.5. Experimental Series 2: Influence of Fitness Evaluation Techniques

From the previous experimental series, we noticed that DE-CNN takes a long time to select the optimal configuration. That is due to the usage of 5-fold CV accuracy as a fitness evaluation technique in DE evaluation stage for each individual. In this experimental series, we analyze the effect of using 80/20 dataset split as the default fitness evaluation technique rather than using 5-fold CV. Tables 6 and 7 depict the classification accuracy of different selected optimal configurations when using 80/20 dataset split. After selecting the optimal configuration, 5-fold CV is applied to reevaluate and assess the generalization of the resulted configuration. In this experimental series, the accuracy and the time cost are the main criteria to select the optimal configuration.

According to the results in Tables 6 and 7, it can be noticed that all experiments nearly have the same accuracy values for each dataset. However, the accuracy when running DE algorithm with the following parameters: DE/best/2 strategy, *F*=0.3, and population size equal to 5 is the best. Moreover, when comparing with the time results from previous experimental series, it can be concluded that adopting 5-fold CV requires more computation time to find the optimal configuration for each dataset which is almost three times compared to 80/20 dataset split. Moreover, the classification accuracy in this case is better than the case were 5-fold CV is used. In contrast, the results for 80/20 dataset split technique are not reliable since random splitting is performed. Therefore, we performed 5-fold CV to reevaluate each selected configuration.

Table 8 depicts the optimal configuration produced for each dataset using DE/best/2, the population of size 5, *F*=0.3, and *CR*=0.2 as DE parameters with 80/20 dataset split. We refer to this model as DE-CNN-TSF.

### 5.6. Experimental Series 3: Influence of DE Algorithm Crossover Probability

In this section, the influence of crossover probability used as a parameter in DE algorithm is analyzed. Two new crossover probability values were chosen, which are 0.5 and 0.8. Conducted experiments in this section were implemented using same setups as in experimental series 2, where *F*=0.3, population of sizes 5 or 10, DE/Best/1 and DE/Best/2. Besides, 80/20 dataset split is used during the search of the optimal configuration. Moreover, 5-fold CV method is applied to the generated optimal configuration for reevaluation, and the 5-fold CV accuracy is reported as the final result in each experiment as shown in Tables 9 and 10 for DE/Best/1 and DE/Best/2, respectively.

From Table 9, it can be concluded that applying a crossover probability *CR*=0.8, a better average accuracy and shorter computational time can be reached. Furthermore, by comparing the two different population sizes at *CR*=0.8, it can be observed that the population of size 5 has better results in both average accuracy and average computation time. Moreover, setting crossover probability *CR* to 0.8 can provide higher classification accuracy and less computation time than setting *CR* to 0.5. As well as, the population of size five can be more accurate and faster than the population of size ten which takes almost twice the computational time compared to the population of size five.

From this experimental series, we can conclude that the best DE-CNN model is constructed at *CR*=0.8, *F*=0.3, population size 5, and DE/best/1 strategy. The model takes a short time to search for the optimal configuration with the highest accuracy values over all different *CR* values. We refer to this model as DE-CNN-TSC.  Table 11 lists optimal configuration of DE-CNN-TSC model for each dataset.

### 5.7. Experimental Series 4: Building a General Model

**Figure 4:**
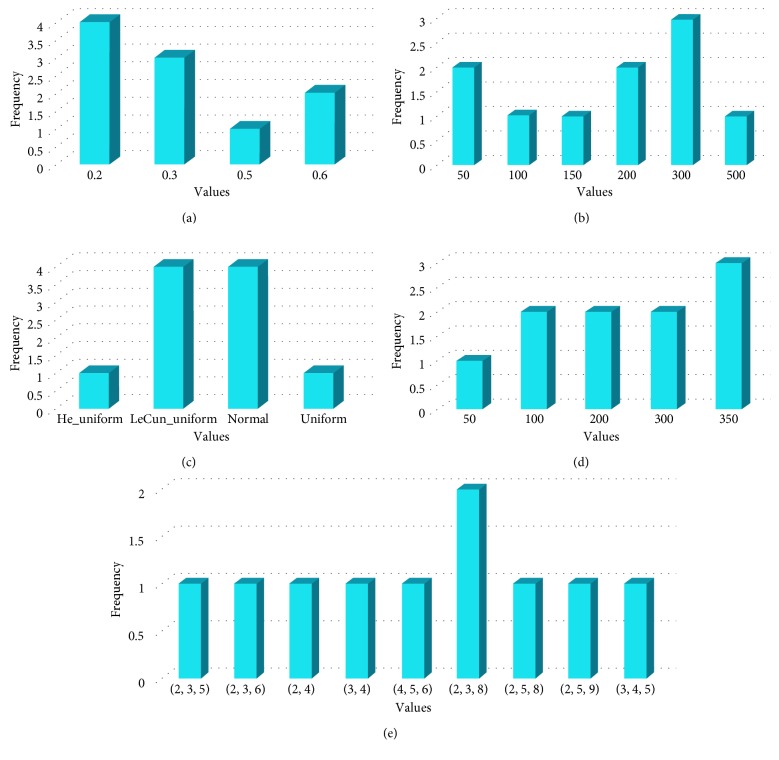
The frequency for each value with the best parameters over all datasets after combining DE-CNN-5CV and DE-CNN-TSC parameters. (a) Dropout rate, (b) NFCS, (c) initialization mode, (d) number of neurons, and (e) filter sizes list.

**Figure 5:**
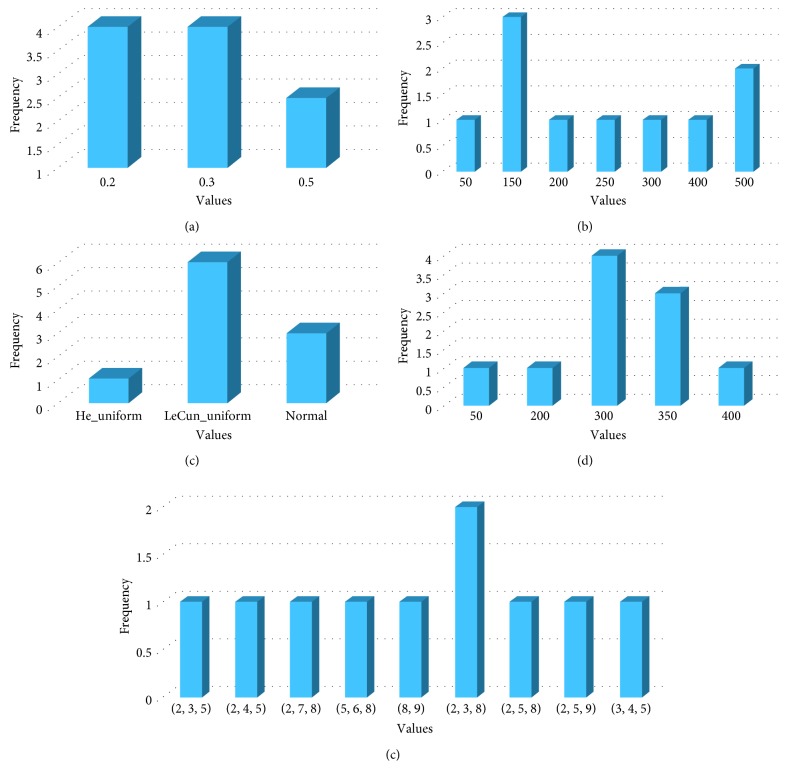
The frequency for each value with the best parameters over all datasets after combining DE-CNN-TSF and DE-CNN-TSC parameters. (a) Dropout rate, (b) NFCS, (c) initialization mode, (d) number of neurons, and (e) filter sizes list.

In this experimental series, a general model only having one optimal configuration is selected for all datasets. The general model is constructed by selecting the most frequent parameters extracted from all optimal configurations during all previous experimental series. We conducted previously three experimental series where we selected the best DE-CNN model that produces the optimal configuration for each dataset. From experimental series 1, 2, and 3, we selected DE-CNN-5CV (*CR*=0.2, *F*=0.3, and DE/best/2), DE-CNN-TSF (*CR*=0.2, *F*=0.3, and DE/best/2), and DE-CNN-TSC (*CR*=0.8, *F*=0.3, and DE/best/2) as best DE-CNN models that produce optimal configuration based on the average classification accuracy and computation time.  Figure 4 shows the frequency of common parameter values when combining DE-CNN-5CV and DE-CNN-TSC. Whereas, Figure 5 shows the frequency of common parameter values of DE-CNN-TSF and DE-CNN-TSC combined.

From Figures 4 and 5, the most frequent value for each parameter will be taken to generate the general model for each combination. However, some parameter values have the same frequency as shown in Figure 4, where two initialization modes (LeCun uniform and normal) have the same frequency which is equal to 4. For that, two general models will be built from each combination based on initialization modes from the first combination (DE-CNN-5CV and DE-CNN-TSC) and dropout rate from the second combination (DE-CNN-TSF and DE-CNN-TSC).  Table 12 lists the configurations of the four generated general models based on the most frequent parameter values extracted from the two different DE-CNN models combinations.

In order to determine the optimal general model from these four models listed in Table 12, they are evaluated on the same datasets using the 10-fold CV. The resulted classification accuracies are reported in Table 13. It can be noticed that the accuracy average of DE-CNN-G1 over all datasets is better than that of the other three models. Moreover, it took less time compared to the other models. Therefore, this model is selected to be the proposed general model.

### 5.8. Experimental Series 5: Comparing with Other Metaheuristic Methods

In this section, the performance of DE algorithm is compared against two metaheuristic methods which are particle swarm optimization (PSO) [55] and genetic algorithm (GA) [56] to search for the optimal parallel CNN model. PSO was trained using parameters such as *ϕ*=2 and *ω*=0.5, while GA trained using a crossover probability equal to 0.5 and a mutation rate equal to 0.3. This comparison is performed using two population sizes which are 5 and 10 for five generations, where 80/20 split technique is used in each metaheuristic algorithm. After generating the optimal configuration using each metaheuristic algorithm, 5-fold and 10-fold evaluation methods are used to assess the general performance of each configuration. Experimental results are given in Table 14.

From Table 14, it can be concluded that the performance of the three algorithms in terms of average classification accuracy is almost the same. However, the DE algorithm has the highest average accuracies and the shortest computation time compared to PSO and GA.

### 5.9. Experimental Series 6: Comparing with State-of-the-Art Methods

In this section, the results of the proposed DE-CNN models are compared with state-of-the-art methods on different Arabic sentiment classification datasets. For a fair comparison with the state-of-the-art methods, the optimal selected configuration from each experimental series is used to build the CNN, where each CNN is evaluated using 10-fold CV, and the results are listed in Table 15. State-of-the-art methods used in the comparison are listed in Table 15.

- CNN-base: a CNN similar to the model described in Section 3.2 trained on Twitter word embeddings (Twt-CBOW) from [58]. A random configuration is used, where parameters such as filter sizes list, number of neurons, NFCS, initialization mode, and dropout rate were set to [3, 5, 7], 150, 100, uniform, and 0.7, respectively.
- Combined LSTM: a model proposed by Al-Azani and El-Alfy [57], where two long short-term memory (LSTM) networks were combined using different combination methods including: summation, multiplication, and concatenation.
- Stacking ensemble (eclf14): a model based on stacking ensemble presented in [21], where several classifiers were included in the training. The used ensemble-learning techniques are stochastic gradient descent (SGD) and nu-support vector classification (NuSVC).
- NuSVC: a model is employed in [7] as a classifier on AAQ dataset.
- SVM(bigrams): a suport vector machine classifier trained on TF-IDF as weighting scheme through bigrams was evaluated in [52] on AJGT dataset.

From Table 15, it can be concluded that DE-CNN-5CV and DE-CNN-G1 are the best models followed by DE-CNN-TSC when comparing in terms of classification accuracy using 10-fold CV for Artwitter dataset. However, there is a small difference between them, but the performance of our three models is better than the performance of other methods on the Artwitter dataset. For STD dataset, DE-CNN-TSF has the highest accuracy value compared to the other two models. For ASTD-B dataset, DE-CNN-G1 model has the best configuration that allows it to possess the highest accuracy among other models. Concerning AJGT and AAQ datasets, DE-CNN-TSC got the highest accuracy among other models. Moreover, regarding F1 measure, DE-CNN-G1 has better values for ArTwitter and ASTD-B datasets. Moreover, the DE-CNN-TSC has better F1 measure values in case of STD, AAQ, and AJGT datasets. Meanwhile, based on the recall measure, DE-CNN-G1 results in highest values for ArTwitter, AAQ, and ASTD-B datasets while the DE-CNN-TSC has the highest recall value for STD dataset. For AJGT dataset, the DE-CNN5CV and DE-CNN-G1 have the same recall value which is the highest value compared to other models recall values.

**Figure 6:**
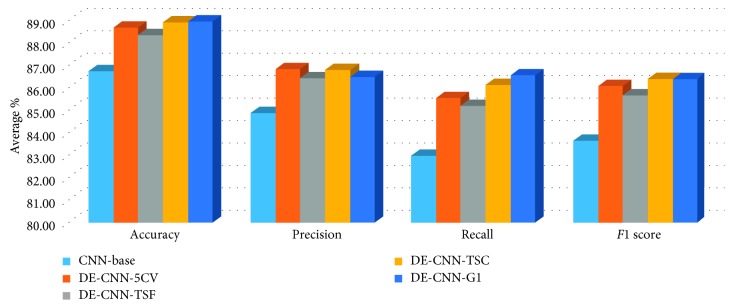
Average of results for the methods on various datasets.

Moreover, the average performance results over all datasets for DE-CNN models and CNN-base model are depicted in Figure 6, where it can be concluded that the best model is the DE-CNN-G1 over all performance measures except at precision where the DE-CNN-TSC performs better.

From all the previous results, we can conclude that the proposed DE-CNN-G1 is the best model over all the other models in this study, where the DE algorithm finds the optimal configuration to build the proper CNN model used to improve Arabic sentiment classification. Moreover, after analyzing DE parameters influence, it has been found that crossover at 0.8, mutation parameters at 0.3, and DE/best/2 improved the ability of DE to find the optimal CNN configuration. With all this accuracy improvement and computational time saving, the proposed DE-CNN in general and DE-CNN-G1 in specific still need to be improved since finding the optimal parameters for the metaheuristic algorithm such as mutation, population size, and the DE strategy require more exploration and exploitation. One of the major conclusions that can be drawn from the results obtained is that the measuring technique of the fitness function value is crucial to the exploration of the architecture and parameters search space of the CNN. Moreover, training a deep neural network usually relies on randomness to perform better. Various forms of randomness can be applied in different stages when training the network such as random initialization of the network weights, setting a regularization using dropout, and during the optimization phase. This phenomenon may affect the stability and repeatability of the obtained results on different evaluation techniques such as 5-fold CV and 10-fold CV.

## 6. Conclusion

This paper proposed a framework that adopts a differential evolution (DE) algorithm for evolving the convolutional neural network (CNN) and generating an Arabic sentiment classification system. The DE mutation strategies help the system by slightly increasing the performance in terms of accuracy and time cost. To further assess the stability of the proposed framework, we built and evaluated two general DE-CNN models using the most frequent parameters extracted from the all optimal configurations. Simulation results show that the two DE-CNN models DE-CNN-TSC and DE-CNN-G1 are robust and stable, and they can outperform state-of-the-art methods.

According to the promising results of the proposed DE-CNN model for enhancing the Arabic sentiment classification, in the future work, the proposed model can be extended and applied to several applications such as image classification, object detection, and big data classification.

## Figures and Tables

**Table 1:** Example of a random configuration *x*_1_.

Parameters	Index range		Example value	
	Minimum	Maximum	Index	*x*_1*j*_ value
Filters sizes list (*x*_11_)	1	20	2	[3, 4]
Number of neurons (*x*_12_)	1	7	4	300
NFCS (*x*_13_)	1	8	3	150
Initialization mode (*x*_14_)	1	4	1	Uniform
Dropout rate (*x*_15_)	1	7	5	0.7

**Table 2:** Individual structure (configuration).

Parameter	Values
*Convolution layer*	
Filter sizes	2 to 9
NFCS	50, 100, 150, 200, 250, 300, 400, 500
*Fully connected layer*	
Number of neurons	50, 100, 200, 300, 350, 400, 500
Initialization mode	Uniform, LeCun uniform, normal, He uniform
Dropout rate	0.2 to 0.9

**Table 3:** The results based on 5-fold CV accuracy as fitness function value used with DE/best/1, different population sizes, and mutation parameter.

DE parameters	DE/best/1-*F*=0.3-*CR*=0.2				DE/best/1-*F*=0.7-*CR*=0.2			
Population size	5		10		5		10	
Dataset	Accuracy	Time (s)	Accuracy	Time (s)	Accuracy	Time (s)	Accuracy	Time (s)
ArTwitter	91.79	7585.98	91.58	22699.37	90.34	13523.39	91.68	22544.00
STD	86.75	4047.13	86.97	8578.22	86.63	10524.26	86.97	17972.58
AAQ	86.03	26251.50	86.10	56413.17	86.12	38387.81	86.73	66221.46
ASTD-B	80.60	6200.27	81.11	5690.58	80.98	4678.05	81.11	9112.36
AJGT	85.75	5061.12	88.56	7862.54	90.83	8486.48	91.72	18209.64
**Average**	86.18	9829.2	86.87	20248.78	86.98	15119.99	**87.64**	**26812.01**

**Table 4:** The results based on 5-fold CV accuracy as fitness function value used with DE/best/2, different population sizes, and mutation parameter.

DE parameters	DE/best/2-*F*=0.3-*CR*=0.2				DE/best/2-*F*=0.7-*CR*=0.2			
Population size	5		10		5		10	
Dataset	Accuracy	Time (s)	Accuracy	Time (s)	Accuracy	Time (s)	Accuracy	Time (s)
ArTwitter	91.48	16570.82	91.27	21842.62	91.37	12498.83	91.73	20115.25
STD	86.63	4160.50	87.03	8306.28	86.75	5956.65	86.75	9726.51
AAQ	85.54	14978.24	86.76	68793.32	83.86	22016.83	85.91	76816.73
ASTD-B	80.85	4408.58	81.48	8175.77	81.04	4455.77	82.05	9056.05
AJGT	91.06	6314.36	92.56	8543.75	92.06	3259.03	91.67	21229.50
**Average**	87.11	9286.5	**87.82**	**23132.35**	87.01	9637.42	87.62	27388.81

**Table 5:** The optimal configuration for each dataset based on the DE-CNN-5CV model.

Dataset	Filter sizes list	Number of neurons	NFCS	Initialization mode	Dropout rate
ArTwitter	[2, 3, 5]	350	50	LeCun uniform	0.6
STD	[2, 4]	100	200	Normal	0.6
AAQ	[4, 5, 6]	200	300	Normal	0.2
ASTD-B	[3, 4]	100	100	He uniform	0.3
AJGT	[2, 3, 6]	200	300	Uniform	0.2

**Table 6:** The results of DE/best/1 strategy, different population sizes, and mutation parameter using 80/20 dataset split reevaluated using 5-fold CV.

DE parameters	DE/best/1-*F*=0.3-*CR*=0.2				DE/best/1-*F*=0.7-*CR*=0.2			
Population size	5		10		5		10	
Dataset	Accuracy	Time (s)	Accuracy	Time (s)	Accuracy	Time (s)	Accuracy	Time (s)
ArTwitter	92.72	2341.01	93.44	3921.61	92.66	3270.71	92.61	6540.61
STD	87.97	1198.57	88.30	2643.58	88.42	3497.13	86.91	5547.04
AAQ	86.97	4949.27	86.38	11575.05	87.43	7880.75	87.39	17957.25
ASTD-B	82.04	1057.19	81.17	1940.99	82.29	1170.57	81.61	2114.62
AJGT	91.67	2858.29	92.56	5039.01	92.50	3382.44	92.44	5338.89
**Average**	88.27	2480.87	88.37	5024.05	88.66	3840.32	88.29	7499.68

**Table 7:** The results of DE/best/2, different population sizes, and mutation parameter using 80/20 dataset split reevaluated with 5-fold CV.

DE parameters	DE/best/2-*F*=0.3-*CR*=0.2				DE/best/2-*F*=0.7-*CR*=0.2			
Population size	5		10		5		10	
Dataset	Accuracy	Time (s)	Accuracy	Time (s)	Accuracy	Time (s)	Accuracy	Time (s)
ArTwitter	92.72	1780.57	92.97	2716.75	92.36	3462.59	92.56	4735.10
STD	88.19	1302.01	87.69	2023.61	88.03	3117.11	88.14	3102.14
AAQ	87.29	9051.87	87.11	20436.34	86.99	7233.76	86.43	20061.08
ASTD-B	81.29	1140.01	82.23	1961.08	81.67	1152.20	82.48	1847.88
AJGT	92.44	3064.20	92.17	6451.25	92.50	2723.33	92.83	6598.21
**Average**	**88.39**	**3267.73**	88.43	6717.81	88.31	3537.80	88.49	7268.88

**Table 8:** The optimal configuration for each dataset based on the DE-CNN-TSF model.

Dataset	Filter sizes list	Number of neurons	NFCS	Initialization mode	Dropout rate
ArTwitter	[2, 3, 5]	200	150	LeCun uniform	0.2
STD	[2, 4, 5]	300	500	LeCun uniform	0.3
AAQ	[8, 9]	350	250	LeCun uniform	0.3
ASTD-B	[5, 6, 8]	300	150	He uniform	0.2
AJGT	[2, 7, 8]	400	400	Normal	0.5

**Table 9:** The results of DE/best/1, different population sizes, and crossover probability using 80/20 dataset split reevaluated with 5-fold CV.

DE parameters	DE/best/1-*F*=0.3-*CR* = 0.5				DE/best/1-*F*=0.3-*CR* = 0.8			
Population size	5		10		5		10	
Dataset	Accuracy	Time (s)	Accuracy	Time (s)	Accuracy	Time (s)	Accuracy	Time (s)
ArTwitter	92.25	1861.73	93.03	3154.44	92.46	700.90	92.56	928.86
STD	88.31	1253.39	87.86	3548.95	88.36	383.54	87.69	730.12
AAQ	87.36	8404.20	87.88	12614.47	87.50	4009.01	87.55	7515.25
ASTD-B	81.54	1313.47	81.79	2041.57	82.17	1271.83	81.85	2756.65
AJGT	93.00	4424.83	92.50	6963.73	92.83	277.75	92.11	780.7900
**Average**	88.49	3451.52	88.61	5664.63	**88.66**	**1328.61**	88.35	2542.33

**Table 10:** The results of DE/best/2, different population sizes, and crossover probability using 80/20 dataset split reevaluated with 5-fold CV.

DE parameters	DE/best/2-*F*=0.3-*CR* = 0.5				DE/best/2-*F*=0.3-*CR* = 0.8			
Population size	5		10		5		10	
Dataset	Accuracy	Time (s)	Accuracy	Time (s)	Accuracy	Time (s)	Accuracy	Time (s)
ArTwitter	92.56	1855.46	93.03	4105.46	93.03	747.39	92.51	1590.83
STD	87.47	2927.36	87.47	6936.65	88.03	444.72	87.64	3682.39
AAQ	87.39	8774.22	87.48	11471.57	87.39	6166.45	87.57	7590.81
ASTD-B	81.98	1015.00	81.48	2619.95	81.85	2330.10	82.10	3463.05
AJGT	92.39	3783.20	92.56	7449.43	92.67	636.44	92.44	1340.59
**Average**	88.36	3671.05	88.40	6516.61	88.59	2065.02	88.45	3533.53

**Table 11:** The optimal configuration for each dataset based on the DE-CNN-TSC model.

Dataset	Filter sizes list	Number of neurons	NFCS	Initialization mode	Dropout rate
ArTwitter	[2, 5, 9]	50	200	LeCun uniform	0.2
STD	[3, 4, 5]	300	300	LeCun uniform	0.3
AAQ	[2, 5, 8]	300	500	Normal	0.5
ASTD-B	[2, 3, 8]	350	150	LeCun uniform	0.3
AJGT	[2, 3, 8]	350	50	Uniform	0.2

**Table 12:** General models configurations.

Combined DE-CNN models	Generated model name	Filter sizes list	Number of neurons	NFCS	Initialization mode	Dropout rate
DE-CNN-TSF and DE-CNN-TSC	DE-CNN-G1	[2, 3, 8]	300	150	LeCun uniform	0.2
	DE-CNN-G2	[2, 3, 8]	300	150	LeCun uniform	0.3
DE-CNN-5CV and DE-CNN-TSC	DE-CNN-G3	[2, 3, 8]	350	300	Normal	0.2
	DE-CNN-G4	[2, 3, 8]	350	300	LeCun uniform	0.2

**Table 13:** Comparison between general models using 10-fold CV.

Dataset	DE-CNN-G1		DE-CNN-G2		DE-CNN-G3		DE-CNN-G4	
	Accuracy	Time (s)	Accuracy	Time (s)	Accuracy	Time (s)	Accuracy	Time (s)
ArTwitter	93.28	552.24	93.23	723.64	92.77	1061.17	93.13	1254.12
STD	88.14	458.10	87.75	486.06	87.97	728.43	88.14	1034.33
AAQ	87.50	1601.06	87.97	1610.54	87.69	2452.71	87.50	2434.58
ASTD-B	82.48	368.28	82.29	185.35	82.60	873.89	82.41	785.69
AJGT	93.06	690.82	92.61	699.20	92.94	1318.60	92.83	1285.41
**Average**	**88.89**	**734.10**	88.77	740.96	88.79	1286.96	88.80	1358.83

**Table 14:** Comparison between DE, PSO, and GA.

Dataset	DE			GA			PSO		
	Time (s)	5-Fold CV	10-Fold CV	Time (s)	5-Fold CV	10-Fold CV	Time (s)	5-Fold CV	10-Fold CV
ArTwitter	700.90	92.46	92.61	767.73	92.41	92.92	1326.14	92.82	92.61
STD	383.54	88.36	88.25	1122.77	87.03	87.80	2079.96	87.25	88.14
AAQ	4009.01	87.50	88.02	6969.59	87.41	87.78	3698.91	87.13	86.89
ASTD-B	1271.83	82.17	82.29	1693.62	81.85	81.73	683.22	82.17	82.29
AJGT	277.75	92.83	93.17	2320.46	92.33	92.72	1122.90	92.44	92.33
**Average**	**1328.61**	**88.66**	**88.87**	2574.83	88.21	88.59	1782.23	88.36	88.45

**Table 15:** Comparisons with other models.

Dataset	Measures	Our models					State-of-the-art models
		CNN-base	DE-CNN-5CV	DE-CNN-TSF	DE-CNN-TSC	DE-CNN-G1	Combined LSTM [57]
ArTwitter	Acc	90.95	93.28	92.25	92.61	93.28	87.27
	Prc	89.76	93.30	90.85	91.24	92.14	87.36
	Rec	93.03	93.74	94.45	94.75	95.05	87.27
	F1	91.32	93.44	92.57	92.91	93.55	87.28

STD		CNN-base	DE-CNN-5CV	DE-CNN-TSF	DE-CNN-TSC	DE-CNN-TS-G1	Stacking (eclf14) [21]
	Acc	87.24	88.31	88.36	88.25	88.14	85.28
	Prc	80.26	79.66	80.41	79.07	79.09	61.04
	Rec	64.82	71.58	70.69	72.25	71.36	67.14
	F1	71.39	75.33	75.14	75.36	74.93	63.95

AAQ		CNN-base	DE-CNN-5CV	DE-CNN-TSF	DE-CNN-TSC	DE-CNN-G1	NuCSV [7]
	Acc	84.69	87.15	87.43	88.01	87.50	80.21
	Prc	83.62	87.38	86.75	88.08	86.70	83.00
	Rec	86.48	87.05	88.49	88.07	88.77	76.50
	F1	85.00	87.16	87.58	88.03	87.70	79.62

ASTD-B		CNN-base	DE-CNN-5CV	DE-CNN-TSF	DE-CNN-TSC	DE-CNN-G1	Combined-LSTM [57]
	Acc	80.47	81.60	80.72	82.28	82.48	81.63
	Prc	81.08	81.89	82.18	82.66	81.86	82.32
	Rec	79.72	81.35	78.84	81.85	83.48	81.63
	F1	80.27	81.54	80.32	82.17	82.57	81.64

AJGT		CNN-base	DE-CNN-5CV	DE-CNN-TSF	DE-CNN-TSC	DE-CNN-G1	SVM (bigrams) [52]
	Acc	90.16	92.72	92.56	93.17	93.06	88.72
	Prc	89.62	91.80	91.86	92.79	92.36	92.08
	Rec	91.00	93.89	93.44	93.67	93.89	84.89
	F1	90.24	92.81	92.63	93.19	93.10	88.27

## Data Availability

The resources used to support the findings of this study have been deposited in the Github repository (https://github.com/dahouabdelghani).
